# Influence of non-nucleoside reverse transcriptase inhibitors (efavirenz and nevirapine) on the pharmacodynamic activity of gliclazide in animal models

**DOI:** 10.1186/1758-5996-1-15

**Published:** 2009-10-09

**Authors:** SK Mastan, K Eswar Kumar

**Affiliations:** 1 Pharmacology Division, AU College of Pharmaceutical Sciences, Andhra University, Visakhapatnam-530 003, Andhra Pradesh, India; 2 Department of Pharmacology, Vignan Institute of Pharmaceutical Technology, Duvvada, Gajuwaka, Visakhapatnam-530 046, Andhra Pradesh, India

## Abstract

**Background:**

Type 2 diabetes may occur as a result of HIV infection and/or its treatment. Gliclazide is a widely used drug for the treatment of type 2 diabetes. Efavirenz and nevirapine are widely used non-nucleoside reverse transcriptase inhibitors for the treatment of HIV infection. The role of Efavirenz and nevirapine on the pharmacodynamic activity of gliclazide is not currently known. The objective of this study was to examine the effect of oral administration of efavirenz and nevirapine on blood glucose and investigate their effect on the activity of gliclazide in rats (normal and diabetic) and rabbits to evaluate the safety and effectiveness of the combination.

**Methods:**

Studies in normal and alloxan induced diabetic rats were conducted with oral doses of 2 mg/kg bd. wt. of gliclazide, 54 mg/kg bd. wt. of efavirenz or 18 mg/kg bd. wt. of nevirapine and their combination with adequate washout periods in between treatments. Studies in normal rabbits were conducted with 5.6 mg/1.5 kg bd. wt. of gliclazide, 42 mg/1.5 kg bd. wt. of efavirenz or 14 mg/1.5 kg bd. wt. of nevirapine and their combination given orally. Blood samples were collected at regular time intervals in rats from retro orbital puncture and by marginal ear vein puncture in rabbits. All the blood samples were analysed for blood glucose by GOD/POD method.

**Results:**

Efavirenz and nevirapine alone have no significant effect on the blood glucose level in rats and rabbits. Gliclazide produced hypoglycaemic/antidiabetic activity in normal and diabetic rats with peak activity at 2 h and 8 h and hypoglycaemic activity in normal rabbits at 3 h. In combination, efavirenz reduced the effect of gliclazide in rats and rabbits, and the reduction was more significant with the single dose administration of efavirenz than multiple dose administration. In combination, nevirapine has no effect on the activity of gliclazide in rats and rabbits.

**Conclusion:**

Thus, it can be concluded that the combination of efavirenz and gliclazide may need dose adjustment and care should be taken when the combination is prescribed for their clinical benefit in diabetic patients. The combination of nevirapine and gliclazide was safe. However, further studies are warranted.

## Background

The study of mechanisms of drug interactions is of much value in selecting the drug concentrations to provide rational therapy. The drug interaction studies assume much importance especially for drugs that have narrow margin of safety and where the drugs are used for prolonged period of time. Diabetes mellitus is one such metabolic disorder that needs treatment for prolonged periods and maintenance of normal blood glucose level is very important in this condition, since both hyperglycemia as well as hypoglycemia is unwanted phenomenon [1].

Diabetes mellitus is a chronic metabolic disorder characterized by elevated blood glucose levels and disturbances in carbohydrate, fat and protein metabolism and an increased risk of complications from vascular disease [2]. Type-1 diabetes is due to decrease in the synthesis of insulin and type-2 diabetes is characterized by hyperglycemia in the context of insulin resistance and relative insulin deficiency. There are estimated 143 million people world wide sufferings from diabetes [3] and the number may probably double by the year 2030 [4]. In India the prevalence rate of diabetes is estimated to be 1-5%.

Among the many metabolic perturbations that occur as a result of Human Immuno Deficiency Virus (HIV) infection and its treatment, alterations in normal glucose homeostasis remain a particularly prevalent and alarming clinical change in affected patients [5]. Much of concern is due to the recognition of the long-term complications of insulin resistance and hyperglycemia and understood is the context of the growing worldwide epidemic of type 2 diabetes mellitus [6].

Insulin resistance, impaired glucose tolerance and type 2 diabetes are conditions that are increasingly described in HIV-1 infected subjects receiving highly active antiretroviral therapy (HAART). HAART generally includes nucleoside reverse transcriptase inhibitors and protease inhibitors. Since many studies have suggested that PI therapy [7] is linked to the development of metabolic complications, it is of importance to propose therapeutic strategies with fewer side effects, such as the use of the non-nucleoside reverse transcriptase inhibitors (NNRTIs) and this approach appear successful to control HIV infection [8].

Efavirenz and nevirapine are NNRTIs used widely in combination with other antiretroviral drugs to treat HIV-infected patients. Recent reports demonstrated that a switch from a protease inhibitor to nevirapine or efavirenz results improvement of metabolic complications in HIV-infected patients [9] and short-term improvement in insulin resistance has been demonstrated with the substitution of nevirapine or efavirenz for the PI component of an antiretroviral regimen [10-12]. However the effect of efavirenz and nevirapine in diabetic condition/oral hypoglycemic agents is unknown.

Oral hypoglycemic agents are used in the treatment of type-2 diabetes, among which gliclazide, a second generation sulphonylurea derivative is preferred in therapy because of its selective inhibitory activity towards pancreatic K^+ ^ATP channels [13-15], antioxidant property [16-18], low incidence of producing severe hypoglycemia [19,20] and other haemobiological effects [21,22]. Gliclazide is known to act mainly by releasing insulin by blocking K^+ ^channels in the pancreatic β cells [23].

Since there is every possibility for the combined use of gliclazide and NNRTIs (efavirenz and nevirapine) in chronic diabetics with associated HIV infection, the study is planned to investigate the effect of efavirenz and nevirapine on blood glucose and their effect on the activity of gliclazide in rats (normal and diabetic) and rabbits to evaluate the safety and effectiveness of the combination with respect to blood glucose level.

## Methods

Gliclazide and NNRTIs (efavirenz and nevirapine) are the gift samples from Micro Labs (Bangalore, India) and Aurobindo Pharma Ltd (Hyderabad, India), respectively. Alloxan monohydrate was purchased from LOBA Chemie (Mumbai, India). Glucose kits (Span diagnostics) were purchased from local pharmacy. All other reagents/chemicals used were of analytical grade.

Albino rats of either sex of 6 to 7 weeks of age, weighing between 250-320 g and normal albino rabbits of either sex of 3 months of age, weighing between 1.35-1.75 Kg were used in the study. They were procured from National Institute of Nutrition, Hyderabad, India. They were maintained under standard laboratory conditions at an ambient temperature of 25 ± 2°C and 50 ± 15% relative humidity with a 12-h light/12-h dark cycle. Animals were fed with a commercial pellet diet (Rayan's Biotechnologies Pvt Ltd., Hyderabad, India) and water *ad libitum*. They were fasted for 18 h prior to the experiment and during the experiment they were withdrawn from food and water. The animal experiments were performed after prior approval of the study protocol by the Institutional Animal Ethics Committee and by the Government regulatory body for animal research. (Reg. No. 516/01/A/CPCSEA). The study was conducted in accordance with the guidelines provided by Committee for the Purpose of Control and Supervision of Experiments on Animals (CPCSEA).

### Study design

In clinical practice, NNRTIs and gliclazide in therapeutic dose will be administered orally as antiretroviral and antidiabetic therapy, respectively. Hence, human oral therapeutic doses of the respective drugs were extrapolated to rat/rabbit based on body surface area [24]. But the dose of gliclazide for rat experiments was selected as 2 mg/kg bd. wt based on the influence of dose-effect relationship of gliclazide on blood glucose in normal rats. Efavirenz and nevirapine were suspended in 0.5% CMC for oral administration [25,26]. Gliclazide solution was prepared by dissolving it in a few drops of 0.1 N NaOH then made up to the volume with distilled water.

The study consists of two phases.

Phase-1: pharmacodynamic interaction study between efavirenz and gliclazide

Phase-2: pharmacodynamic interaction study between nevirapine and gliclazide

Each phase consists of 3 stages.

Stage-1: study in normal rats

Stage-2: study in diabetic rats

Stage-3: study in normal rabbits

### Study in normal rats

A group of six rats was administered with 2 mg/kg bd. wt of gliclazide, orally. The same group was administered with interacting drug (efavirenz 54 mg/kg bd. wt. or nevirapine 18 mg/kg bd. wt., orally) and the combination of interacting drug and gliclazide. One week washout period was maintained between treatments. After this single dose interaction study the same group was continued with the daily treatment of interacting drug (efavirenz/nevirapine) for the next eight days with regular feeding. Later after 18 h fasting they were again given the combined treatment on the ninth day.

### Study in diabetic rats

Diabetes was induced in rats by the administration of alloxan monohydrate in two doses, i.e. 100 mg and 50 mg/kg bd. wt intraperitoneally for two consecutive days [27]. After 72 h, samples were collected from rats by orbital puncture of all surviving rats and the serum was analyzed for glucose levels. Rats with blood glucose levels of 200 mg/dl and above were considered as diabetic and selected for the study. The same treatment as described in the study in normal rats was performed with a group of six alloxan-induced diabetic rats.

### Study in normal rabbits

A group of six rabbits was administered with 5.6 mg/1.5 kg bd. wt of gliclazide, orally. The same group was administered with interacting drug (efavirenz 42 mg/1.5 kg bd. wt. or nevirapine 14 mg/1.5 kg bd. wt., orally) and the combination of efavirenz and gliclazide. One week washout period was maintained between treatments. After this single dose interaction study the same group was continued with the daily treatment of interacting drug (efavirenz/nevirapine) for the next eight days with regular feeding. Later after 18 h fasting they were again given the combined treatment on the ninth day.

### Collection of blood samples

Blood samples were withdrawn from retro orbital plexus [28] of each rat at 0, 1, 2, 3, 4, 6, 8 and 12 h. Blood samples were withdrawn from the marginal ear vein of each rabbit at 0, 1, 2, 3, 4, 6, 8, 12, 16, 20 and 24 h. These blood samples were analysed for blood glucose by GOD/POD method [29] using commercial glucose kits.

### Data and statistical analysis

Data were expressed as mean ± SEM. The significance was determined by applying Student's paired 't' test.

## Results

### Pharmacodynamic interaction study between efavirenz and gliclazide

Gliclazide produced hypoglycemic activity with maximum biphasic reduction of 41.64 ± 0.80% and 38.70 ± 1.43% at 2 h and 8 h respectively in normal rats (Table 1). Gliclazide produced antihyperglycemic activity with maximum biphasic reduction of 42.05 ± 1.73% and 44.05 ± 1.55% at 2 h and 8 h respectively in diabetic rats (Table 2). Gliclazide produced hypoglycemic activity with maximum reduction of 34.25 ± 0.99% at 3 h in normal rabbits (Table 3). Efavirenz alone has not produced any significant effect on the blood glucose level of rats (normal and diabetic) and rabbits (Tables 1, 2, 3). In combination efavirenz has reduced the gliclazide activity in rats and rabbits and the reduction was more significant with the single dose treatment of efavirenz than multiple dose treatment (Tables 1, 2, 3).

**Table 1:** Mean percent blood glucose reduction in normal rats (N = 6)

**Time** **(h)**	**Gliclazide**	**Efavirenz**	**Efavirenz + Gliclazide (Single dose treatment)**	**Efavirenz + Gliclazide (Multiple dose treatment)**
1	31.46 ± 1.39	-06.66 ± 2.95	24.40 ± 1.20***	26.57 ± 1.09*
2	41.64 ± 0.80	-04.44 ± 1.44	33.77 ± 0.87**	36.26 ± 1.53*
3	28.21 ± 0.95	-02.94 ± 0.96	21.19 ± 1.15***	22.26 ± 0.99**
4	24.21 ± 1.13	-00.06 ± 1.80	16.16 ± 0.79***	18.68 ± 0.96***
6	31.47 ± 1.46	02.14 ± 1.49	23.34 ± 1.09***	28.71 ± 1.85
8	38.70 ± 1.43	04.29 ± 1.09	28.36 ± 1.70***	31.21 ± 2.03**
10	26.08 ± 1.02	06.86 ± 1.78	19.00 ± 1.44***	20.45 ± 1.11**
12	12.27 ± 1.55	06.13 ± 1.59	08.27 ± 0.64	08.98 ± 1.02

***Significant at P < 0.001 compared to gliclazide control

**Significant at P < 0.01 compared to gliclazide control

*Significant at P < 0.05 compared to gliclazide control

**Table 2:** Mean percent blood glucose reduction in diabetic rats (N = 6)

**Time** **(h)**	**Gliclazide**	**Efavirenz**	**Efavirenz + Gliclazide (Single dose treatment)**	**Efavirenz + Gliclazide (Multiple dose treatment)**
1	32.92 ± 1.95	-04.55 ± 2.55	24.83 ± 1.98**	28.26 ± 1.62
2	42.05 ± 1.73	-05.08 ± 2.46	30.02 ± 1.59***	36.39 ± 1.89*
3	31.21 ± 1.74	-03.21 ± 2.41	20.70 ± 1.31***	25.45 ± 1.93*
4	25.13 ± 1.77	-02.40 ± 2.24	16.78 ± 2.21***	20.78 ± 1.36*
6	36.14 ± 1.56	03.99 ± 1.97	22.15 ± 1.34***	30.12 ± 1.47*
8	44.05 ± 1.55	04.45 ± 2.26	32.01 ± 1.39***	38.27 ± 1.42*
10	28.56 ± 1.87	05.39 ± 1.72	16.25 ± 2.25***	20.89 ± 1.49**
12	24.66 ± 2.32	04.28 ± 1.53	08.49 ± 2.32***	11.31 ± 2.17***

***Significant at P < 0.001 compared to gliclazide control

**Significant at P < 0.01 compared to gliclazide control

*Significant at P < 0.05 compared to gliclazide control

**Table 3:** Mean percent blood glucose reduction in normal rabbits (N = 6)

**Time** **(h)**	**Gliclazide**	**Efavirenz**	**Efavirenz + Gliclazide (Single dose treatment)**	**Efavirenz + Gliclazide (Multiple dose treatment)**
1	18.30 ± 1.88	-04.36 ± 1.49	09.25 ± 1.37***	11.54 ± 1.24***
2	24.50 ± 1.24	-06.16 ± 1.93	14.96 ± 1.12***	16.61 ± 1.21***
3	34.25 ± 0.99	-03.63 ± 1.24	24.97 ± 0.87***	27.14 ± 0.67***
4	25.92 ± 1.30	-01.48 ± 1.22	14.61 ± 1.22***	16.97 ± 1.40***
6	24.50 ± 0.96	-00.04 ± 0.98	12.44 ± 1.59***	14.05 ± 1.53***
8	18.96 ± 2.85	02.09 ± 1.94	10.69 ± 0.70***	12.28 ± 0.80**
12	10.39 ± 1.32	04.22 ± 2.31	08.89 ± 1.23	10.78 ± 1.86
16	05.71 ± 1.13	06.03 ± 1.87	04.59 ± 1.35	06.51 ± 0.54
20	04.24 ± 1.54	05.31 ± 2.28	03.17 ± 1.29	03.87 ± 2.26
24	02.08 ± 1.47	04.60 ± 1.78	01.42 ± 0.45	02.85 ± 0.90

***Significant at P < 0.001 compared to gliclazide control

**Significant at P < 0.01 compared to gliclazide control

### Pharmacodynamic interaction study between nevirapine and gliclazide

Gliclazide produced hypoglycemic activity with maximum biphasic reduction of 40.72 ± 0.56% and 37.46 ± 1.18% at 2 h and 8 h respectively in normal rats (Table 4). Gliclazide produced antihyperglycemic activity with maximum biphasic reduction of 42.50 ± 1.40% and 44.46 ± 1.46% at 2 h and 8 h respectively in diabetic rats (Table 5). Gliclazide produced hypoglycemic activity with maximum reduction of 36.18 ± 1.08% at 3 h in normal rabbits (Table 6). Nevirapine alone has not produced any significant effect on the blood glucose level of rats (normal and diabetic) and rabbits (Tables 4, 5, 6). In combination nevirapine has no impact on the gliclazide activity in rats and rabbits following single and multiple dose treatments (Tables 4, 5, 6).

**Table 4:** Mean percent blood glucose reduction in normal rats (N = 6)

**Time** **(h)**	**Gliclazide**	**Nevirapine**	Nevirapine + Gliclazide*(Single dose treatment)	Nevirapine + Gliclazide*(Multiple dose treatment)
1	30.88 ± 1.12	-01.44 ± 0.46	30.43 ± 1.09	30.83 ± 0.94
2	40.72 ± 0.56	02.88 ± 0.45	38.35 ± 0.72	40.49 ± 0.84
3	28.34 ± 0.82	03.96 ± 0.64	26.84 ± 0.79	28.30 ± 0.68
4	23.97 ± 0.79	04.68 ± 0.65	22.90 ± 0.99	23.63 ± 1.13
6	30.90 ± 0.46	02.51 ± 0.66	30.42 ± 1.00	31.53 ± 0.67
8	37.46 ± 1.18	01.43 ± 0.71	35.14 ± 1.23	37.62 ± 1.44
10	24.73 ± 0.69	01.05 ± 1.66	23.61 ± 1.01	25.81 ± 0.76
12	10.52 ± 1.14	00.34 ± 1.40	10.33 ± 1.30	11.80 ± 1.16

*Statistically no significance compared to gliclazide control

**Table 5:** Mean percent blood glucose reduction in diabetic rats (N = 6)

**Time** **(h)**	**Gliclazide**	**Nevirapine**	Nevirapine + Gliclazide*(Single dose treatment)	Nevirapine + Gliclazide*(Multiple dose treatment)
1	33.45 ± 1.76	02.08 ± 0.38	32.42 ± 1.47	33.23 ± 1.63
2	42.50 ± 1.40	04.34 ± 1.40	41.68 ± 1.27	42.28 ± 1.32
3	31.87 ± 1.68	04.42 ± 1.62	30.69 ± 1.61	31.66 ± 1.66
4	25.88 ± 1.42	06.11 ± 1.61	25.43 ± 1.47	25.82 ± 1.34
6	36.89 ± 1.30	06.28 ± 1.53	36.25 ± 1.48	36.78 ± 1.38
8	44.46 ± 1.46	03.08 ± 0.54	43.25 ± 1.35	44.37 ± 1.33
10	28.72 ± 1.76	01.67 ± 0.67	27.93 ± 1.55	28.92 ± 1.68
12	24.39 ± 1.81	01.64 ± 0.71	23.81 ± 1.80	24.98 ± 1.83

*Statistically no significance compared to gliclazide control

**Table 6:** Mean percent blood glucose reduction in normal rabbits (N = 6)

**Time** **(h)**	**Gliclazide**	**Nevirapine**	Nevirapine + Gliclazide*(Single dose treatment)	Nevirapine + Gliclazide*(Multiple dose treatment)
1	19.44 ± 2.25	02.08 ± 2.38	21.09 ± 2.93	20.39 ± 2.00
2	26.73 ± 2.57	03.57 ± 2.48	25.38 ± 2.40	26.56 ± 0.90
3	36.18 ± 1.08	05.72 ± 2.23	34.38 ± 0.69	35.18 ± 1.39
4	28.90 ± 3.23	04.59 ± 2.66	26.48 ± 1.47	27.61 ± 1.83
6	24.62 ± 2.00	02.53 ± 1.89	23.21 ± 2.51	25.49 ± 1.05
8	19.13 ± 2.46	02.50 ± 1.42	18.59 ± 1.80	19.63 ± 2.79
12	14.46 ± 1.76	02.82 ± 2.51	12.87 ± 0.86	12.49 ± 1.91
16	08.23 ± 1.85	01.00 ± 2.29	06.36 ± 2.31	07.14 ± 0.99
20	04.99 ± 1.49	01.04 ± 1.54	03.92 ± 1.52	05.33 ± 1.18
24	02.14 ± 1.09	00.63 ± 1.94	01.37 ± 1.43	03.87 ± 1.91

*Statistically no significance compared to gliclazide control

## Discussion

HIV infected patients are likely to suffer with diabetes mellitus [5] and hence most often antiretroviral drugs are co-administered along with oral antidiabetic drugs. HIV infection and diabetes are both chronic diseases that significantly affect lifestyle. When they intersect, the treatment regimens required for both diseases can be overwhelming for patients. Frequently prescribed antiretroviral drugs belong to the class of non-nucleoside reverse transcriptase inhibitors (NNRTIs) in HIV-infected patients.

Efavirenz and nevirapine are commonly prescribed NNRTIs for the treatment of HIV-infection and known to be improving the metabolic complications in HIV-infected patients [9-12]. However, there is no much evidence on the activity of efavirenz/nevirapine alone in diabetic condition, as well as their effect on the activity of gliclazide. Based on these factors the study was planned to investigate the effect of efavirenz/nevirapine on blood glucose and its effect on the activity of gliclazide in rats (normal and diabetic) and rabbits to evaluate the safety and effectiveness of the combination with respect to blood glucose level. In our study, the multiple dose effect of efavirenz and nevirapine on the gliclazide activity was also studied for the influence of the long term treatment with efavirenz/nevirapine since both are used for chronic period.

Drug interactions are usually seen in clinical practice and the mechanisms of interactions are evaluated usually in animal models (rodents and non-rodents). We studied the influence of efavirenz and nevirapine on the activity of gliclazide in rats (normal and diabetic) and rabbits. The normal rat model served to quickly identify the interaction and diabetic rat model served to validate the same response in the actually used condition of the drug. The rabbit model is another dissimilar species to validate the occurrence of the interaction. Since small amount of blood was required for glucose analysis, the blood samples were collected by retro-orbital puncture as it was reported to be good method when small samples of blood were required [28]. Diabetes was induced with alloxan monohydrate, since it was more economical and easily available.

Gliclazide produced biphasic response in rat model when administered alone, which may be due its biliary excretion and entero hepatic cycling [30]. Such effect is not seen in rabbit model. Gliclazide is known to produce hypoglycemic/antihyperglycemic activity by pancreatic [31-33] (stimulating insulin secretion by blocking K^+ ^channels in the pancreatic *β *cells) and extra pancreatic [34-36] (increasing tissue uptake of glucose) mechanisms.

Our study revealed the safety profile of efavirenz and nevirapine in diabetic condition also, with respect to blood glucose levels. However, contrary to the theoretical expectation, the activity of gliclazide was reduced in the presence of efavirenz in rats (normal and diabetic) and rabbits and it confirms the presence of potential interaction between efavirenz and gliclazide. The impact of efavirenz on the activity of gliclazide was more significant following single dose administration.

*In vitro *studies have shown that efavirenz inhibits CYP2C9, CYP2C19 and CYP3A4 with inhibition constant (K_i_) values (8.5-17 μM) in the range observed efavirenz plasma concentrations [37,38]. *In vitro and in vivo *studies also demonstrated that efavirenz induces CYP3A4 activity in a concentration- and time-dependent manner [25,39,40]. Clinical drug-drug interaction studies showed that efavirenz decreased the systemic exposure of several CYP3A4 substrates, such as amprenavir, indinavir and methadone [41-43] in addition to the CYP2C9 and CYP2C19 substrates [44]. Gliclazide is known to be metabolized by hepatic microsomal enzymes CYP2C9 primarily and partly by CYP3A4 [33,45]. So the decreased activity of gliclazide in the presence of efavirenz may be due to its increased metabolism by hepatic microsomal enzymes. During initial preclinical studies, chronic administration of efavirenz was shown to induce its own metabolism and to increase activities of CYP3A4 in rats, rhesus monkeys, and humans [39,46,47]. So this autoinduction may be the reason behind the less impact of the efavirenz on the activity of gliclazide following multiple dose administration in rats and rabbits. Overall the interaction between gliclazide and efavirenz appears to be due to pharmacokinetic rather than pharmacodynamic in nature. The present study is limited to describe the exact mechanism of action(s) behind this interaction and it has to be confirmed by conducting pharmacokinetic interaction studies in different species.

On other side, nevirapine is known to be an inducer of CYP3A4 and CYP2B6 [48,49]. There is a minor and non-significant reduction in gliclazide activity following nevirapine administration in rats and rabbits. The minor and non-significant decreased activity of gliclazide in the presence of nevirapine may be due to its increased metabolism by hepatic microsomal enzyme CYP3A4. Even though the reduction in gliclazide activity in the presence of nevirapine is minor and non-significant, comparatively the reduction is more with single dose treatment of nevirapine than multiple dose treatment. Just like efavirenz, nevirapine is also known to be undergoing autoinduction of CYP3A4 and CYP2B6 mediated metabolism following multiple dose administration [50]. So the effect associated with the multiple dose treatment of nevirapine may be due to autoinduction of nevirapine. However, either the single dose or multiple dose treatment of nevirapine has no significant impact on the gliclazide activity in rats (normal and diabetic) and rabbits and it confirms the combination was safe with respect to blood glucose level.

## Conclusion

Since the interaction between efavirenz and gliclazide was seen in two dissimilar species, it is likely to occur in humans also leading to decreased activity of gliclazide, which may need dosage adjustment. Hence care should be taken when the combination is prescribed for their clinical benefit in diabetic patients. Since there is no interaction between nevirapine and gliclazide in any species, it is likely to be safe combination in humans also. However the present study warrants further studies to find out the relevance of these interactions in human beings and to know the exact mechanism of action behind this interaction(s) if any.

## List of abbreviations

CMC: Carboxymethyl cellulose; CYP: Cytochrome P-450; GOD/POD: Glucose oxidase peroxidase; HIV: Human immuno deficiency virus; K^+ ^ATP: Potassium adenosine triphosphate; NNRTIs: Non-nucleoside reverse transcriptase inhibitors; PIs: Protease inhibitors.

## Competing interests

The authors declare that they have no competing interests.

## Authors' contributions

SKM Participated in the design, carried out the study and drafted the manuscript.

KEK Conceived of the study, participated in the design of the study and performed the statistical analysis and interpretation of the data.

Both the authors read and approved the final manuscript.
